# Effect of PREDICT on chemotherapy/trastuzumab recommendations in HER2-positive patients with early-stage breast cancer

**DOI:** 10.3892/ol.2014.2589

**Published:** 2014-10-07

**Authors:** SUE K. DOWN, OLIVIA LUCAS, JOHN R. BENSON, GORDON C. WISHART

**Affiliations:** 1 Cambridge Breast Unit, Addenbrooke’s Hospital, Cambridge, UK; 2 Faculty of Health, Social Care and Education, Anglia Ruskin University, Cambridge, UK

**Keywords:** breast cancer, management, prognostic factors, erbB-2 receptor, online predictive tools, age

## Abstract

PREDICT is an online prognostication tool for early-stage breast cancer, which incorporates human epidermal growth factor 2 (HER2) status and stratifies absolute treatment benefits for hormone therapy, chemotherapy and trastuzumab. The present study compared historical multidisciplinary team (MDT) decisions regarding adjuvant treatment with PREDICT estimates, to determine whether certain patients are being over- or undertreated, particularly when stratified by age and oestrogen-receptor (ER) status. HER2-positive early-stage breast cancer cases over a five-year period at the Cambridge Breast Unit (Addenbrooke’s Hospital, Cambridge, UK) were retrospectively reviewed. Patients receiving neo-adjuvant therapy were excluded. Adjuvant chemotherapy/trastuzumab recommendations based on PREDICT (<3%, no benefit; 3–5%, discuss treatment; and >5%, recommend treatment) were compared with actual MDT decisions. In total, 109 eligible patients were identified. The average age at diagnosis was 59.6 years, with 21 patients older than 70 years (19%). Four patients were predicted to gain an absolute benefit of >5% from chemotherapy/ trastuzumab, but were not offered treatment (all >70 years). Amongst the 19 patients aged >70 years predicted to benefit >3%, six were not offered treatment (32%). In the patients aged <69 years, there was evidence of overtreatment with adjuvant chemotherapy/trastuzumab in 8 out of 12 cases with <3% benefit using PREDICT. For all 20 patients with ER-negative tumours, the MDT and PREDICT decisions correlated, whilst for ER-positive cases, more than half (8 out of 14) were offered treatment despite a <3% predicted benefit. PREDICT can aid decision-making in HER2-positive early-stage breast cancer by identifying older patients at risk of undertreatment with chemotherapy/trastuzumab, and by reducing the overtreatment of patients with little predicted benefit, particularly in ER-positive disease.

## Introduction

In early-stage breast cancer, accurate prognostication and the analysis of treatment benefit is important in selecting the appropriate adjuvant therapy following surgery. Treatment decisions traditionally made by multidisciplinary teams (MDTs) can now be aided by predictive models based on large patient cohorts. A variety of validated tools are available that provide estimates of survival and treatment benefit.

Initial prognostication tools have provided survival estimates within defined prognostic groups. The Nottingham Prognostic Index (NPI), originally developed in 1982 and updated in 2007, was derived from data on symptomatic patients in the UK, pre-dating the screening era (1,2). The NPI incorporates prognostic factors derived from surgical pathology, including tumour grade, size and lymph node status, to provide 10-year survival estimates within specified prognostic groups.

Adjuvant! (www.adjuvantonline.com/index.jsp) was introduced in 2001 and is a free web-based tool for use by health care professionals, based on data from the USA Surveillance Epidemiology and End Results Program (3). Adjuvant! provides individualised estimates of 10-year survival and treatment benefits for hormone therapy and chemotherapy, in addition to calculating the risk of disease relapse. Unlike the NPI, the Adjuvant! model incorporates age, co-morbidities and oestrogen receptor (ER) status, in addition to post-operative pathological data, and has been validated in cohorts from British Columbia and the Netherlands (4,5). Nonetheless, Adjuvant! does have certain limitations; in a UK validation study, Adjuvant! was shown to overestimate overall survival by 6% (6). In addition, Adjuvant! does not include the method of cancer detection nor human epidermal growth factor 2 (HER2) status in the calculation of survival estimates.

PREDICT (www.predict.nhs.uk) is an alternative freely available web-based breast cancer prognostication and treatment benefit tool for use by health professionals and the public, which was introduced in 2010 (7). The original model is based on a cohort of 5,694 females diagnosed in the UK between 1999 and 2003, and has been validated in an independent UK case cohort and a British Columbia data set, where performance was comparable with Adjuvant! (8). PREDICT initially incorporated patient age, ER status and standard pathological prognostic factors, in addition to mode of detection, which is an independent prognostic and predictive factor (9). PREDICT provides estimates of overall survival and treatment benefit from hormone therapy, chemotherapy and trastuzumab at 5- and 10-year time-points.

Since the initial development of these tools, further molecular factors have been recognised to be important in breast cancer prognostication. Human epidermal growth factor receptor 2 (HER2) is an oncogene amplified in up to 30% of breast cancers and associated with a poorer prognosis and higher likelihood of recurrence following initial treatment (10). Trastuzumab is a monoclonal antibody therapy targeted against the HER2 receptor, which is administered in combination with chemotherapy in either adjuvant or neo-adjuvant regimens. Side-effects include cardiotoxicity, and careful monitoring of cardiac function is required throughout treatment (usually a 12-month course). Several trials have studied the efficacy of trastuzumab in early-stage HER2-positive breast cancer, and have shown benefits in disease-free and overall survival, with ~50% reduction in disease recurrence (B31/N9831, BCIRG006, FinHER and HERA) (10–13).

PREDICT was updated in October 2011 to incorporate the prognostic effect of HER2 status, using data from >10,000 patients in the Breast Cancer Association Consortium (14). The updated model (PREDICT Plus) was validated by comparing 10-year survival estimates, using the original PREDICT and Adjuvant! tools, with actual 10-year outcomes from the British Columbia data set (comprising 1,653 patients: HER2-positive, n=203; HER2-negative, n=1,450). In patients with HER2-positive breast cancer, the updated PREDICT tool provided better estimates of overall survival and breast cancer-specific survival than the other two models, emphasising the importance of HER2 status in prognostication (14).

Breast cancer is increasingly recognised as a spectrum of disease with tumour heterogeneity requiring individualised treatment regimes. Moreover, patients are increasingly involved in the clinical decision-making process and require clearly presented personalised data to aid them. Tools which estimate prognosis do not necessarily predict which tumours will respond well to adjuvant chemotherapy, leading to over- and undertreatment of early-stage breast cancers. Several commercial tests have been developed that provide more accurate predictions of response to chemotherapy, including gene expression profiling (Oncotype DX, Mammaprint) and expanded immunohistochemical analysis (NPI Plus) (15). However, these tests are costly and not currently recommended by the National Institute for Health and Clinical Excellence outside of research protocols. In the current economic climate, the cost of such technologies must be carefully balanced against the benefits achieved in terms of survival and quality of life.

At present, the majority of patients with HER2-positive tumours receive chemotherapy and trastuzumab, regardless of their ER status. However, adjuvant chemotherapy is associated with a greater benefit in reduction of disease recurrence for ER-negative tumours (16). Therefore, certain patients with both HER2- and ER-positive tumours may be overtreated, thus causing unnecessary morbidity for little overall survival advantage. Although the use of prognostication tools is becoming more widespread in routine clinical practice, the absence of a model including HER2 status prior to PREDICT Plus has necessitated subjective judgement in MDTs to determine the optimal adjuvant therapy for HER2-positive cases.

The aim of the present study was to compare historical MDT decision-making for adjuvant therapy with novel estimates derived from PREDICT to determine whether clinical judgement alone is effective, and whether certain patient groups with early-stage HER2-positive disease are being over- or undertreated, particularly when stratified by age and ER status. Elucidation of the most accurate prognostication and treatment benefit techniques in this patient group is clearly important, as up to one-quarter of early-stage breast cancer diagnoses are HER2-positive (17).

## Patients and methods

### Clinical decision-making

Prior to October 2011, the combination of adjuvant chemotherapy and trastuzumab was routinely recommended for all patients presenting to the Cambridge Breast Unit (Addenbrooke’s Hospital, Cambridge, UK) with HER2-positive tumours, unless they had significant co-morbidities. Since October 2011, the PREDICT absolute 10-year survival benefit from chemotherapy has been routinely used in the MDT decision-making process as follows: <3%, chemotherapy not recommended; 3–5%, chemotherapy discussed as a treatment option; and >5%, chemotherapy recommended. These values were selected to maximise the benefit to risk ratio of chemotherapy treatment, and to ensure that individual patient variables were taken into account.

### Patient cohort

This study includes all females presenting to the Cambridge Breast Unit with HER2-positive early-stage breast cancer diagnosed between January 2007 (when routine testing of HER2 status commenced) and October 2011 (when the updated PREDICT model became available). Exclusion criteria included evidence of metastatic disease at presentation, patients treated in the private sector (problems with access to records) and patients receiving neo-adjuvant therapy.

### Comparator recommendations

Calculated adjuvant chemotherapy/trastuzumab recommendations based on PREDICT treatment benefits were compared with MDT decisions recorded contemporaneously.

### Statistical analysis

Statistical analysis was performed using Fisher’s exact testing and Microsoft Excel (Microsoft Corporation, redmond, WA, USA). P<0.05 was used to indicate a statistically significant difference.

## Results

### Eligible group

Over the study period of 57 months, 186 females with HER2-positive early-stage breast cancer were treated in the public sector. In total, 77 patients received neo-adjuvant chemotherapy and trastuzumab, and were therefore excluded from this study. Therefore, 109 patients were eligible for inclusion.

### Patient characteristics

A total of 69 cases presented symptomatically (63%), with the remainder detected by routine breast screening. Patient and tumour characteristics are summarised in Table I.

The average patient age was 60 years, with a median age of 60 years and 21 patients aged ≥70 years (19%). Tumours were generally of high grade (III) and ER-positive.

### Surgical treatment

All patients underwent primary surgical treatment with successful breast conserving surgery (n=56 cases; 51%) or mastectomy (n=53 cases; 49%). Axillary surgery was undertaken for all cases, except three who refused any surgical staging procedure. Almost two-thirds of patients underwent sentinel lymph node biopsy as the only axillary procedure (n=64 cases; 59%), with the remaining 42 patients requiring axillary node clearance due to nodal involvement detected pre-operatively with core needle biopsy, or as a completion procedure due to at least one sentinel node containing macro- (>2 mm) or micro-metastases (0.2–2 mm).

### Chemotherapy recommendations

A comparison of the MDT decision regarding adjuvant chemotherapy and trastuzumab, and the PREDICT estimate of treatment benefit is shown in Table II.

The historical MDT decision and the PREDICT estimate was concordant in 91 cases (83%). Six patients were not offered treatment, as the benefit was believed to be minimal, and this was confirmed in all six cases by the PREDICT analysis, which gave a benefit estimate of <3% over 10 years. Chemotherapy and trastuzumab were offered to 85 patients following MDT discussion, and a benefit of >3% over 10 years was confirmed by PREDICT.

### Disconcordant cases

Of the 18 discordant cases (indicated by asterisks in Table II), 10 represented potential undertreatment of the disease, and the remaining eight represented possible overtreatment.

Four patients were predicted to gain an absolute benefit of >5% from chemotherapy and trastuzumab, but were not offered treatment (all were >70 years of age). A further six patients were not offered adjuvant therapy following MDT recommendation, but had a moderate indication of benefit (3–5%) using PREDICT. Overall, 8 patients were offered adjuvant chemotherapy and trastuzumab, although PREDICT estimated the benefit to be <3% at 10 years. All of these patients were aged <70 years (mean, 58 years), and all had ER-positive disease.

In a subgroup analysis of those patients (n=21) aged ≥70 years, the majority were predicted to benefit >3% from adjuvant therapy (n=19; 90%), despite 6 not being offered adjuvant chemotherapy and trastuzumab (32%). Furthermore, when comparing the likelihood of undertreating patients, an age of ≥70 years is a significant risk factor (P=0.0033). In the remaining 88 patients aged <70 years, 12 cases were predicted to benefit <3% from adjuvant treatment (14%), amongst whom, eight were offered chemotherapy and trastuzumab (67%). Nonetheless, age was not a statistically significant factor for overtreatment of the disease (P=0.3495).

### Chemotherapy and ER status

All 19 patients with ER-negative tumours were offered adjuvant chemotherapy and trastuzumab following MDT discussion. This was fully concordant with PREDICT estimates of benefit, which is most likely associated with a paucity of other treatment options. By contrast, of the 90 patients with ER-positive disease, 14 were predicted to gain <3% benefit from adjuvant therapy and eight of these were offered treatment following MDT discussion (57%). However, ER status alone was not an independent risk factor for overtreatment of the disease (P=0.3461).

## Discussion

The results of this retrospective study indicate that MDT decisions regarding adjuvant therapy for HER2-positive early-stage breast cancer without the use of predictive tools are appropriate in the majority of cases. This is conditional upon the MDT comprising an appropriate group of experienced clinicians from relevant disciplines. However, the present study also identified selected groups of patients for whom application of a predictive tool, which includes HER2 status, may improve adjuvant decision-making. These include older patients (≥70 years) who are more likely to be offered suboptimal treatment, and those patients with ER-positive disease whose absolute benefit is less from chemotherapy and trastuzumab due to endocrine therapy sensitivity.

However, there are limitations to the present study. Firstly, the number of patients in certain subgroups was comparatively small (≥70 years and ER-negative disease), which may limit detection of any statistically significant differences between groups. Secondly, the choice of adjuvant therapy was based on factors other than those included in the PREDICT model of calculation, for example, patient co-morbidities. Of the four patients predicted to gain >5% benefit from adjuvant therapy, but who were not offered treatment following MDT discussion, one had significant cardiac morbidity precluding the use of chemotherapy, with a survival time of only 8 months following surgery. However, the remaining three patients, all ≥70 years, did not have any specific contraindication to adjuvant chemotherapy and trastuzumab and their median overall survival time following surgery was 57.7 months. The inclusion of the American Society of Anaesthesiologists grade in PREDICT (as in Adjuvant!) may therefore affect decision-making in only a minority of cases.

In total, 78 patients received adjuvant chemotherapy and trastuzumab, with one-quarter of patients (n=20) experiencing treatment-related morbidity, ranging from nausea to neutropenic sepsis and cardiac dysfunction. The combined use of adjuvant chemotherapy and trastuzumab carries a significant risk of side-effects, which should be borne in mind in patients with ER-positive/HER2-positive early-stage breast cancer that may derive minimal benefit from more aggressive therapy. Decisions may be aided by additional tumour markers that are now available, including the proliferative factor, Ki67, which predicts poorer survival and response to chemotherapy. This latter factor has recently been incorporated into the PREDICT tool and awaits validation (18).

A significant proportion of patients (41%) presenting to the Cambridge Breast Unit with HER2-positive early-stage breast cancer received neo-adjuvant endocrine therapy or chemotherapy on MDT recommendation, and were therefore excluded from the present study. Amongst these 77 patients, 72 received a combination of chemotherapy and trastuzumab, and five received endocrine therapy alone. PREDICT is unable to calculate estimates of survival and treatment benefit for these patients as post-operative pathological tumour size and lymph node involvement are frequently downstaged. Of these 77 neo-adjuvant patients, 21 (27%) were found to have a pathological complete response (pCR) on final histopathological analysis, with no viable tumour remaining. However, it remains a challenge to select subgroups of patients who are most likely to benefit from neo-adjuvant therapy. A recent study of HER2-positive breast cancer treated with neo-adjuvant chemotherapy and trastuzumab suggested that patients with ER-negative disease are more likely to achieve a pCR than those with ER-positive disease (19,20). Patients undergoing neo-adjuvant therapy should be closely monitored to exclude disease progression.

The present study has highlighted the potential impact of age on the type of adjuvant therapy offered, with patients aged ≥70 years significantly less likely to be offered adjuvant chemotherapy and trastuzumab. The contemporary female life expectancy in the UK is 82 years (Office for National Statistics, Sept 2011), with approximately one-third of females presenting with newly diagnosed breast cancer when aged >70 years (21). In the current study, 19% of HER2-positive breast cancer patients were in this older age group. For the period between 2010 and 2011, 7% of screen-detected cancers were detected in females aged >70 years in England (22). The screening age range has now been extended and continued screening can be requested beyond the age of 73 years. Previous studies have documented inequalities in breast cancer treatment amongst older females, with those aged ≥70 years being less likely to be offered surgery and chemotherapy, and having poorer breast cancer relative survival (23,24). Although increasing age is associated with co-morbidities, which may preclude the use of certain adjuvant treatments, a number of older patients are healthy and have a reasonable life expectancy post-surgery. These patients should therefore be considered as potential candidates for adjuvant chemotherapy/trastuzumab treatment.

Notably, although PREDICT was originally derived from data on females aged between 23 and 95 years, it has been shown in validation studies to underestimate 10 year mortality in the >75-years age group (7). As greater numbers of older patients undergo treatment and follow-up for breast cancer, more data will become available to update PREDICT estimates in the ≥70-years age group.

In conclusion, PREDICT is a validated prognostication and treatment benefit tool that can effectively aid decision-making for adjuvant chemotherapy and trastuzumab in HER2-positive early-stage breast cancer, and in turn potentially reduce the over- and undertreatment of the disease. The PREDICT model continues to evolve and most recently includes the tumour maker Ki67. In the future, as more data becomes available, PREDICT will be a particularly useful tool for estimating the benefit from adjuvant treatments in the older patient age group.

## Figures and Tables

**Table I:** Patient and tumour characteristics of the study population of HER2-positive early-stage breast cancer patients diagnosed at the Cambridge Breast Unit between January 2007 and October 2011 (n=109).

Characteristic	Value
Mean patient age (range), years	59.6 (28–83)
Mean tumour size (range), mm	22.8 (3–80)
Tumour grade, n	
Grade 1	1
Grade 2	32
Grade 3	76
LN status, n	
LN-negative	60
1–3 LNs	32
≥4 LNs	14
Unknown	3
ER status, n	
Positive	90
Negative	19

LN, lymph node; ER, oestrogen receptor; HER2, human epidermal growth factor 2.

**Table II:** Comparison of the MDT recommendation and predicted benefit of adjuvant therapy with chemotherapy and trastuzumab in HER2-positive early-stage breast cancer patients diagnosed at the Cambridge Breast Unit between January 2007 and October 2011.

	PREDICT benefit of adjuvant chemotherapy/trastuzumab			
	
MDT recommendation	<3%	3–5%	>5%	Total
No	6	6*	4*	16
Yes	8*	20	65	93

* Discordant cases.

MDT, multidisciplinary team; HER2, human epidermal growth factor 2.
